# Prevalence and factors associated with e-cigarettes consumption among adolescents: A cross-sectional analysis in Latin America and the Caribbean

**DOI:** 10.1371/journal.pgph.0007058

**Published:** 2026-08-11

**Authors:** Paolo J. Taipe-Rivero, Liseth J. Humpire-Mamani, Antonio Bernabé-Ortiz

**Affiliations:** Universidad Científica del Sur, Lima, Perú; Cancer Institute Women's India Association, INDIA

## Abstract

E-cigarette use among adolescents has increased globally; however, there is little evidence about usage patterns and their associated factors in Latin America and the Caribbean. This study aimed to estimate the prevalence and evaluate the factors associated with e-cigarette use among adolescents in 16 Latin American and Caribbean countries. A secondary data analysis was performed using the Global Youth Tobacco Survey collected between 2014 and 2022. Age-standardized prevalences and their respective 95% confidence intervals (95% CI) were calculated, and Poisson regression models were used to identify associated factors, reporting prevalence ratios (PR) and their 95% CIs. Data from 55,330 adolescents were analyzed. The prevalence of e-cigarette use in the past 30 days was 8.0% (95% CI: 6.9%–9.3%), whereas daily use was 0.7% (95% CI: 0.5%–1.1%), with significant differences by sex (p = 0.002). Factors associated with e-cigarette use were older age (PR = 1.29; 95%CI: 1.17-1.41), exposure to tobacco smoke at home (PR = 2.25; 95%CI: 1.46-3.46) and at school (PR = 2.16; 95%CI: 1.77-2.62). On the other hand, daily use was associated with sex (PR = 0.35; 95%CI: 0.17-0.73), spending capacity (PR = 4.01; 95%CI: 1.94-8.27) and exposure to smoking at home (PR = 3.47; 95%CI: 1.54-7.80). Among adolescents in Latin America and the Caribbean, e-cigarette use was significant and depended on the indicator used. Any e-cigarette use was associated with older age, smoking exposure at home and at school, whereas daily e-cigarette use was greater among men, and associated with spending capacity and smoking exposure at home. These findings underscore the need to strengthen public policies that restrict access to these products and promote tobacco- and vaping-free school and home environments.

## Introduction

The use of electronic cigarettes (e-cigarettes or EC) has increased significantly over the last decade, emerging as a major challenge for global public health [1], with potential health complications, including respiratory irritation, lung edema, sustained tissue injury, arterial stiffness, among others. Initially promoted as an aid for smoking cessation, these devices have gained popularity—particularly among adolescents and young adults—even among those who had never consumed conventional tobacco [2,3]. In certain high-income countries, such as the United States, the prevalence of EC use within the last 30 days among middle school students (grades 6–8, mean age: 12.7 years) exceeds 25%. However, this estimate was approximately 10% among high school students (grades 9–12, mean age: 16.1 years) [4]. In Latin America, while the literature remains scarce, a systematic review of 14 studies reported an EC use prevalence among adolescents across six countries ranging from 2.4% in Argentina to 64.2% in Mexico, with a combined mean prevalence of 18.9% [5].

EC use can be assessed using various indicators such as lifetime use (experimentation indicator), use in the last 30 days (recent use indicator), daily use (regular or dependent use), number of daily puffs (intensity), nicotine concentration used, type of device and flavors, and refill method [6,7]. The present study focused on two indicators widely used in epidemiological surveillance: use in the last 30 days and daily use, as they offer complementary perspectives for understanding the magnitude and pattern of use, and have comparable indicators across different countries.

The adolescent group is particularly vulnerable to EC use due to a combination of individual factors, such as the perception of low risk; social factors, including peer pressure and the family environment; and contextual factors, such as advertising, availability, local policies [8,9]. Beyond these elements, international studies have demonstrated that the prevalence of EC use varies considerably across different countries. This variation is attributed to disparities in regulatory frameworks, product accessibility, social norms, and the effectiveness of prevention strategies [10,11].

In Latin America, evidence on EC use among adolescents is limited and scattered, whereas no information is available from the Caribbean [5]. Some countries have implemented different regulations to regulate EC use. However, many of them regulate EC based on their tobacco classification. Of the existing countries regulating EC, about a third do not have regulation specifically for EC but instead apply existing tobacco control regulation to these products [12]. The Latin America and Caribbean region, with its high sociocultural diversity, represents a key setting for studying how EC use varies across national contexts, and what individual or environmental factors might be associated with it [5,13].

In this context, the present study aimed to estimate the prevalence and analyze the factors associated with the consumption of EC (in its recent and daily forms) among adolescents from 16 countries in Latin America and the Caribbean, using comparable data collected through the Global Youth Tobacco Survey (GYTS).

## Materials and methods

### Study design

A secondary analysis was conducted using data from GYTS, a survey developed to monitor tobacco use and its determinants among adolescents. This cross-sectional, population-based survey is administered to students aged 12–16 and employs a standardized methodology, allowing for valid comparisons between countries and regions [14]. For this analysis, data collected between 2014 and 2022 in 16 countries of Latin America and the Caribbean were used. However, some major nations (i.e., Brazil, Mexico, Colombia and Chile) do not participate in the GYTS, which may affect representativeness and limit the extrapolation of our results.

For participant selection, a multistage random sampling method was used. The first phase consisted of selecting schools as primary sampling units and classrooms as secondary sampling units, with students present at the time of the survey being eligible to participate [15]. This type of study is justified by the representative, cross-sectional, and comparable nature of the data, and because it allows for the exploration of relevant trends and associations at the population level, especially in contexts where access to national surveys is limited.

### Survey characteristics

The GYTS was developed by the Centers for Disease Control and Prevention (CDC) in collaboration with the World Health Organization (WHO) [14]. Survey data are publicly accessible and available on the official WHO platform [16]. The questionnaire includes 50–70 questions organized into thematic modules that address: prevalence of tobacco use (conventional and electronic), exposure to secondhand smoke, attitudes toward tobacco use, access, advertising exposure (tobacco products on TV, social media, etc.), knowledge about the risks of tobacco, and school-based behaviors (schoolmate smoking, teacher smoking, etc.).Although the sampling methodology is standardized and applied uniformly across all participating countries, the specific set of questions may vary according to the priorities and sociocultural characteristics of each country. This flexibility allows the survey to be adapted to the national context without compromising its international methodological comparability [15].

### Variables definition

The variables of interest were two: (a) e-cigarette use during the last 30 days and (b) daily e-cigarette use during the last 30 days. Both variables were obtained through the question, “During the last 30 days, how many days did you use e-cigarettes?” The response options were: 0, 1, or 2; 3–5; 6–9; 10–19; 20–29; and 30 days. Therefore, for the first variable of interest, those who reported using e-cigarettes at least once in the last month were considered to have used e-cigarettes in the last 30 days (yes vs. no), whereas for the second variable, those who reported using e-cigarettes for all 30 days were considered to have used EC daily (yes vs. no). Given this latter definition is very restrictive, we also included an extra definition for daily EC use during the last 30 days: 20 or more days within the past 30 days as reported in other studies in a more conservative way (sensitivity analysis).

In addition, variables such as age (in years) and sex (male and female), self-reported according to the GYTS survey, were used. A variable related to spending capacity was also included, obtained through the question, “During an average week, how much money do you have to spend on yourself as you wish?”. The response options had different ordered categories (i.e., from “Do not have any spending money” to greater categories [less than 5 PEN, or greater than 50 PEN in the case of Peru]). Given the use of different currencies (studies from various countries) and the diversity in the year the surveys were administered, it was decided that those who responded having no money to spend would be compared with those who reported having it (yes vs. no), regardless of the amount reported. For the variable of passive exposure to tobacco smoke at home, the question “During the last 7 days, how many days has someone smoked inside your home in your presence?” was used, where those who answered “0 days” were compared with those who reported being exposed at least one day a week (yes vs. no). The variable of exposure to tobacco in school was assessed by asking “During the last 30 days, did you see anyone smoking inside the school building or outside of school property?” and the response options (yes vs. no) were used for analysis. Finally, the survey year (from 2014 to 2022) was also evaluated.

### Statistical analysis

The analysis was performed using STATA 16 software (StataCorp, College Station, TX, US). A complex sampling design was considered, incorporating strata, clusters, and weights. In addition, the subpopulation approach was applied for analyses stratified by sex and country [17].

The study population was described using mean and standard deviation (SD) for numerical variables, and absolute and relative frequencies for categorical variables. Age-standardized prevalences were calculated using the WHO standard population (age groups: 10–14 and 15–19 years) as a reference [18]. Comparisons between groups were performed using the Rao and Scott second-order corrected chi-square test, suitable for data from complex surveys [19].

Poisson regression models, both bivariate and multivariate, were applied to estimate prevalence ratios (PR) with 95% confidence intervals (95% CI). We opted for Poisson instead of logistic regression because country-specific prevalences in several settings exceeded 10%, and logistic odds ratios would be inflated relative to prevalence ratios in those settings (overestimation bias) [20]. Given the exploratory nature of the analysis, the multivariate model was constructed by including all reported variables in a single model, and only those that were significant (p-value <0.05) were considered relevant for discussion. Because each participating country contributed data from a single survey year, country and survey year were highly collinear; we therefore included country as a fixed effect, which absorbs both unmeasured country-level contextual factors and the time of data collection. Finally, since data spanned eight years (2014 – 2022), a period during which the e-cigarette market, product diversity, regulatory landscape, and social norms around vaping have changed substantially across the region, we conducted a sensitivity analysis excluding surveys administered before 2018, the approximate period of rapid market expansion.

### Ethics

The survey data is available free of charge and without personally identifiable information. The protocol for this analysis was evaluated and reviewed by the Ethics Committee of the Universidad Científica del Sur (code: PRE-15-2024-00423).

## Results

### Characteristics of the study population

The studies were conducted between 2014 (Belize) and 2022 (Suriname). Sample sizes ranged from 1,453 in Argentina (2018) to 8,735 in Nicaragua (2019), with a total of 55,330 across the 16 registered countries in Latin America and the Caribbean (see **Table 1**). Of the 55,330 records, 2,390 were not considered because of missing values in e-cigarette variables. Missing values ranged from 0.1% in Peru (2019) to 34.4% in El Salvador (2021).

**Table 1:** Data available for statistical analysis (n = 52,940).

Country	Study year	Sample size	Missing values*	Mean age (SD)	Female (%)
Antigua and Barbuda	2017	2,268	0.7%	14.5 (1.0)	49.0%
Argentina	2018	1,453	5.5%	14.0 (1.0)	50.8%
Belize	2014	1,900	0.6%	14.1 (1.2)	51.6%
Bolivia	2018	5,155	6.8%	14.5 (1.0)	50.0%
Cuba	2018	4,172	0.7%	13.9 (1.2)	49.1%
El Salvador	2021	2,763	34.4%	14.4 (1.0)	49.1%
Jamaica	2017	1,685	1.0%	14.7 (1.0)	55.5%
Nicaragua	2019	8,735	1.1%	13.8 (1.2)	49.7%
Panama	2017	2,621	0.2%	13.7 (1.1)	49.9%
Paraguay	2019	4,698	0.5%	13.3 (1.0)	50.0%
Peru	2019	4,148	0.1%	14.1 (1.3)	49.9%
St Lucia	2017	1,495	1.5%	13.6 (1.1)	49.0%
St Vincent and the Grenadines	2018	1,519	1.8%	14.8 (1.0)	50.5%
Suriname	2022	1,838	2.7%	14.0 (1.3)	52.3%
Trinidad and Tobago	2017	4,128	3.4%	14.1 (1.4)	51.6%
Venezuela	2019	6,752	8.4%	13.8 (1.1)	51.8%

* The missing values only include variables of interest for analysis.

The lowest mean age was 13.3 (SD: 1.0) years in Paraguay, whereas the highest mean age was 14.7 (SD: 1.0) years in Saint Vincent and the Grenadines. The overall proportion of females participating in the study was 50.5%, with the lowest value in Antigua and Barbuda and Saint Lucia (49.0% in both cases) and the highest value in Jamaica (55.5%).

### Prevalence and factors associated with e-cigarette use in the past 30 days

**Fig 1:**
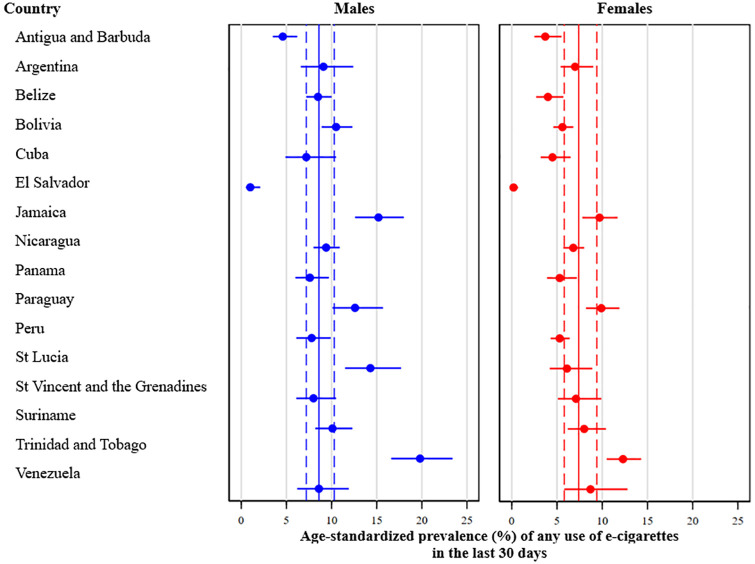
Prevalence of electronic cigarette use during the last 30 days: analysis by country and by sex.

The overall prevalence of e-cigarette use in the past 30 days was 8.0% (95% CI: 6.9%–9.3%), being higher in males (8.6%) than females (7.4%), although this difference was not statistically significant (p = 0.32). The highest prevalence of e-cigarette use in the past 30 days was in Trinidad and Tobago (15.9%, 95% CI: 14.1%–17.9%), whereas the lowest estimate was in El Salvador (0.6%, 95% CI: 0.4%–1.1%). Consistent with this pattern, the prevalence for both sexes was higher in Trinidad and Tobago, whilst the lowest prevalence for both sexes was observed in El Salvador (see Fig 1 and S1 Table).

In a multivariable model, older age, passive exposure to tobacco at home, and exposure to tobacco at school were associated with e-cigarette use in the past 30 days (see Table 2). On the other hand, although females showed a lower prevalence than males, this finding was not statistically significant. Similarly, spending capacity did not show an association. Our sensitivity analysis, excluding surveys before 2018, is in S2 Table, and point estimates and 95% CI were essentially unchanged.

**Table 2:** Factors associated with any and daily use of e-cigarettes: crude and adjusted models.

	Any use of electronic cigarettes in the last 30 days		Daily consumption of electronic cigarettes in the last 30 days	
	Bivariable model	Multivariable model	Bivariable model	Multivariable model
	PR (95% CI)	PR (95% CI)	PR (95% CI)	PR (95% CI)
**Sex**				
Female vs. male	0.86 (0.64 – 1.16)	0.88 (0.58 – 1.31)	**0.38 (0.20 – 0.72)**	**0.35 (0.17 – 0.73)**
**Age**				
For each additional year	**1.33 (1.23 – 1.44)**	**1.29 (1.17 – 1.41)**	1.22 (0.92 – 1.60)	1.25 (0.92 – 1.70)
**Spending capacity**				
Yes vs. no	**1.52 (1.04 – 2.21)**	1.57 (0.97 – 2.54)	**2.22 (1.17 – 4.23)**	**4.01 (1.94 – 8.27)**
**Exposure to smoking at home**				
Yes vs. no	**2.68 (1.86 – 3.86)**	**2.25 (1.46 – 3.46)**	**4.40 (1.83 – 10.53)**	**3.47 (1.54 – 7.80)**
**Exposure to smoking at school**				
Yes vs. no	**2.23 (1.86 – 2.67)**	**2.16 (1.77 – 2.62)**	**2.28 (1.08 – 4.82)**	1.83 (0.98 – 3.43)

PR = Prevalence ratio; 95% CI = 95% confidence intervals.

The multivariable models were also adjusted for country as a fixed effect. PR of this latter variable is not shown (see S3 Table for details).

### Prevalence and factors associated with daily e-cigarette use in the last 30 days

The overall prevalence of daily e-cigarette use in the last 30 days was 0.7% (95% CI: 0.5%–1.1%), with a higher prevalence in males (1.1%) than in females (0.4%), a difference that was statistically significant (p = 0.002). In sensitivity analysis, using the second definition of daily EC use in the last 30 days, the overall prevalence estimate was 0.9% (95%CI: 0.7%–1.3%), with a higher prevalence in males (1.3%) than females (0.5%, p < 0.001).

The highest prevalence of daily e-cigarette use in the last 30 days was observed in Trinidad and Tobago (2.0%, 95% CI: 1.2%–3.3%), whereas the lowest was in Nicaragua (0.0%, 95% CI: 0.0%–0.1%). Consistent with the overall pattern, the highest prevalence in both sexes was in Trinidad and Tobago, whilst the lowest prevalence was in Nicaragua (see Table 3).

**Table 3:** Prevalence of daily electronic cigarette use in the last 30 days: analysis by country and by sex.

Country	Prevalence of daily electronic cigarette use		
	Total	Males	Females
	P (95% CI)	P (95% CI)	P (95% CI)
Antigua and Barbuda	0.3% (0.2% - 0.8%)	0.6% (0.3% - 1.3%)	0.2% (0.0% - 0.8%)
Argentina	0.6% (0.3% - 1.5%)	1.0% (0.4% - 2.7%)	0.2% (0.0% - 2.1%)
Belize	0.6% (0.3% - 1.1%)	1.1% (0.5% - 2.1%)	0.0% (0.0% - 0.8%)
Bolivia	0.5% (0.3% - 0.9%)	0.7% (0.4% - 1.4%)	0.3% (0.1% - 0.6%)
Cuba	0.5% (0.3% - 0.8%)	0.6% (0.3% - 1.3%)	0.5% (0.3% - 0.6%)
El Salvador	0.1% (0.0% - 0.4%)	0.1% (0.0% - 0.6%)	0.0% (0.0% - 0.6%)
Jamaica	0.8% (0.5% - 1.4%)	1.2% (0.6% - 2.3%)	0.3% (0.1% - 0.9%)
Nicaragua	0.0% (0.0% - 0.1%)	0.0% (0.0% - 0.1%)	0.0% (0.0% - 0.1%)
Panama	0.0% (0.0% - 0.4%)	0.0% (0.0% - 0.7%)	0.0% (0.0% - 0.3%)
Paraguay	0.6% (0.4% - 0.9%)	0.9% (0.5% - 1.6%)	0.2% (0.1% - 0.5%)
Peru	0.1% (0.0% - 0.3%)	0.1% (0.0% - 0.5%)	0.1% (0.0%-0.5%)
St Lucia	0.8% (0.4% - 1.4%)	1.3% (0.7% - 2.5%)	0.3% (0.1% - 1.1%)
St Vincent and the Grenadines	0.7% (0.4% - 1.4%)	0.6% (0.2% - 1.5%)	0.9% (0.4% - 2.2%)
Suriname	0.7% (0.3% - 1.3%)	0.4% (0.1% - 1.4%)	0.9% (0.3% - 2.0%)
Trinidad and Tobago	2.0% (1.2% - 3.3%)	3.0% (1.7% - 5.1%)	1.0% (0.5% - 2.1%)
Venezuela	1.1% (0.6% - 1.9%)	1.6% (0.7% - 3.3%)	0.6% (0.4% - 0.9%)

The prevalence of daily e-cigarette use in the past 30 days was higher in males than in females, in those who reported spending capacity, and among those with passive exposure to tobacco at home, but not among those exposed to tobacco at school. However, no significant association was found with age (see Table 2). Our sensitivity analysis, excluding surveys before 2018, is also in S2 Table, and point estimates and 95% CI were essentially unchanged.

## Discussion

### Main findings

This study found that the prevalence of e-cigarette use, known as vaping, among adolescents in Latin America and the Caribbean is highly heterogeneous, varying markedly across countries. Several associated factors were identified, including sex (more frequent in men than women), spending capacity, and exposure to tobacco smoke at home and in schools. Furthermore, although the proportion of adolescents reporting daily e-cigarette use remains low, the prevalence of daily consumption is not trivial, representing a critical public concern towards nicotine dependence and other sequelae [21].

### Comparison with previous studies

The overall prevalence of recent e-cigarette use (in the last 30 days) in our sample was 8%. This estimate is consistent with the global average of 9.2% described in the Global Youth Tobacco Survey in 68 countries and territories [22], and lower than the prevalence reported in some high-income countries, such as the United States, where data from the National Youth Tobacco Survey, conducted in 2020, show that 19.6% of high school students and 4.7% of junior high school students used e-cigarettes in the last 30 days [3].

In our analysis, the countries with the highest prevalence of e-cigarette use in the past 30 days were Trinidad and Tobago (15.9%), Jamaica (12.5%), and Paraguay (11.3%). These values fall within the range reported by a recent regional analysis of Latin American adolescents, which documented prevalences between 2.4% and 64.2%. In different cross-sectional surveys, Mexico (64.2%), Colombia (32.3%), and Puerto Rico (21.9%) showed the highest prevalence of EC use, but these countries do not collect data in the GYTS [5]. This difference may be due to variations in availability, regulation, and marketing standards among Latin American and Caribbean countries.

In addition to the GYTS, several studies show marked regional variability in the use of e-cigarettes and nicotine products among adolescents. In Mexico, recent school surveys have reported a prevalence of e-cigarette use in the last 30 days of between 7% and 10% among middle and high school students [21]. In contrast, in Panama, the GYTS recorded that approximately 7% of adolescent students were current e-cigarette users, showing substantial differences even within the same region [23].

Regarding associated factors, our findings on age, exposure to tobacco smoke at home or school, and access to money are consistent with the international literature, which has consistently shown that older adolescents have higher rates of tobacco use [22], that exposure to parental or school smoke increases the risk of use [5], and that greater financial availability facilitates the acquisition of devices [24]. Furthermore, previous research has identified other determinants not assessed in the GYTS, such as mental health symptoms [25], exposure to social media content [26], low risk perception [27], and the influence of digital marketing and influencers [28]. However, these variables could not be analyzed in this study due to limitations of the questionnaire.

The distinction between occasional and frequent or daily use is fundamental to understanding e-cigarette use patterns among adolescents. Occasional use is often related to curiosity, experimentation, or exposure to advertising [1], whilst frequent use is linked to greater product availability, the presence of friends or parents who use e-cigarettes, and environments where the behavior is normalized [24,26]. More detailed studies have used indicators such as frequency of use, device recharging, nicotine dependence, and motivations for use to characterize these patterns more precisely [25].

At the regional level, scientific evidence in Latin America and the Caribbean remains limited; however, recent studies in Mexico, Brazil, and Uruguay have documented significant prevalence rates of e-cigarette use among adolescents, as well as risk patterns like those observed in other regions [12,21,22]. Nevertheless, differences in methodology, geographic coverage, and type of measurement make direct comparisons between available studies difficult [5]. Moreover, to our knowledge, the lack of intervention studies in the region suggests the need to adapt or use strategies used in high-income countries.

### Relevance of our findings

Our findings underscore the imperative for multi-level preventive frameworks tailored to the adolescent demographic. Evidence-based strategies, such as banning flavored e-cigarette products and the rigorous regulation of digital marketing, have been shown to significantly attenuate nicotine initiation [3,28]. At the micro-systemic level (family), parental education regarding the influence of behavioral modeling and the negative effects of environmental tobacco smoke (ETS) within domestic settings constitute an essential pillar of comprehensive risk mitigation [5].

From a regulatory perspective, whereas 21 countries within the Americas have ratified some form of legislative oversight concerning electronic nicotine delivery systems, a significant regulatory heterogeneity persists: only eight nations have enacted comprehensive sales bans, whereas the majority maintain permissive or fragmented regulatory structures [29]. This legislative heterogeneity may serve as a critical driver of cross-national variation in e-cigarette consumption patterns. Specifically, countries that lack robust restrictions on e-commerce platforms or flavor additives exhibit a positive correlation with greater prevalence rates among adolescents [26].

### Strengths and limitations

Key strengths of this study include the use of nationally representative and comparable data across multiple countries, as well as the standardized GYTS approach, which allows for a robust assessment of the phenomenon in the region. Furthermore, stratified and sensitivity analysis and the use of multivariate models enabled the identification of relevant adjusted associations.

However, this study has several limitations. First, cross-sectional design of surveys prevents establishing causal relationships between the variables. Second, the use of self-reported data introduces the risk of recall bias and social desirability bias [30]. In other more advanced studies, complementary methods such as measuring cotinine in saliva or urine to verify nicotine exposure, cross-validation with peers, or longitudinal follow-up have been used to improve the validity of the results [31,32]. Third, data from El Salvador showed 34% of missing values, which may impact pooled estimates. Thus, we compared results including and excluding data from El Salvador and results varied only 0.1% and non-response was not differentially distributed by sex, but it was by age group. Because non-response in El Salvador was differential by age group, and because e-cigarette use rises with age in our data, the country-specific estimate for El Salvador may be biased downward; pooled estimates were unaffected as confirmed by the sensitivity analysis. Fourth, some temporal inconsistency is found in some variables (i.e., tobacco smoke at home is evaluated over the last 7 days, the exposure at school over the last 30 days, and EC use over the last 30 days). This inconsistency may have effect on some estimates. For example, the shorter reference window for smoke home exposure may have attenuated the point estimate of the association. Fifth, gender diversity was not captured by the GYTS as non-binary or gender-nonconforming adolescents may have distinct patterns of EC use as has been documented in other countries [33]. Sixth, the dichotomization of the spending capacity variable (no vs. yes) may affect our capacity to show a dose-response analysis as we decided for this strategy to pool multi-country, multi-currency data. Finally, the study does not include psychosocial variables such as depressive symptoms, risk perception, social media use, or the educational level of the parents, which could play an important role in e-cigarette consumption among adolescents.

## Conclusions

Among adolescents in Latin America and the Caribbean, e-cigarette use was significant and depended on the indicator used. Thus, about one in twelve adolescents reported e-cigarette use in the past 30 days, with prevalence rates over 10% in Trinidad and Tobago, Jamaica, Paraguay, and St Lucia. This study provides contextual evidence on the factors associated with e-cigarette use among adolescents in Latin America and the Caribbean, differentiating between types of use (occasional and daily) and highlighting how certain individual and environmental factors relate differently to each pattern. The variability observed between countries suggests that regulatory and educational policies should be adapted to local realities, considering that many countries still lack specific regulations or have only partial regulatory frameworks.

## Supporting information

S1 Table Prevalence of any electronic cigarette use in the past 30 days: analysis by country and by sex. (DOCX)

S2 Table Factors associated with any and daily use of e-cigarettes: crude and adjusted models using information from 2018 and onwards. (DOCX)

S3 Table Factors associated with any and daily use of e-cigarettes: crude and adjusted models (considering country coefficients in models). (DOCX)
