# Supplementary material for: Prevalence and factors associated with e-cigarettes consumption among adolescents: A cross-sectional analysis in Latin America and the Caribbean
Source: PLOS Glob Public Health. 2026 Aug 11;6(8):e0007058. doi: 10.1371/journal.pgph.0007058 (PMC13460583; doi:10.1371/journal.pgph.0007058)
Supplement: Supplementary file 1 [file pgph.0007058.s001.docx]

**S1 Table.** Prevalence of any electronic cigarette use in the past 30 days: analysis by country and by sex

| **Country** | **Prevalence of daily electronic cigarette use** | | |
| --- | --- | --- | --- |
|  | **Total** | **Males** | **Females** |
|  | **P (95% CI)** | **P (95% CI)** | **P (95% CI)** |
| Antigua and Barbuda | 4.2% (3.3% - 5.3%) | 4.6% (3.5% - 6.2%) | 3.7% (2.5% - 5.5%) |
| Argentina | 8.0% (6.1% - 10.5%) | 9.1% (6.6% - 12.4%) | 7.0% (5.4% - 9.0%) |
| Belize | 6.1% (5.0% - 7.5%) | 8.5% (7.2% - 10.0%) | 4.0% (2.7% - 5.7%) |
| Bolivia | 8.0% (6.9% - 9.4%) | 10.5% (8.9% - 12.3%) | 5.6% (4.6% - 6.8%) |
| Cuba | 5.9% (4.2% - 8.3%) | 7.2% (4.9% - 10.5%) | 4.5% (3.2% - 6.5%) |
| El Salvador | 0.6% (0.4% - 1.1%) | 1.0% (0.5% - 2.1%) | 0.2% (0.1% - 0.7%) |
| Jamaica | 12.5% (11.0% - 14.1%) | 15.2% (12.6% - 18.0%) | 9.7% (7.8% - 11.7%) |
| Nicaragua | 8.1% (7.0% - 9.2%) | 9.4% (8.0% - 10.9%) | 6.8% (5.7% - 8.0%) |
| Panama | 6.4% (5.1% - 8.2%) | 7.6% (6.0% - 9.7%) | 5.3% (3.9% - 7.2%) |
| Paraguay | 11.3% (10.1% - 12.6%) | 12.6% (10.1% - 15.7%) | 9.9% (8.2% - 11.9%) |
| Peru | 6.5% (5.3% - 8.0%) | 7.8% (6.1% - 9.9%) | 5.3% (4.3% - 6.4%) |
| St Lucia | 10.3% (8.3% - 12.7%) | 14.3% (11.5% - 17.7%) | 6.1% (4.2% - 8.9%) |
| St Vincent and the Grenadines | 7.6% (6.1% - 9.4%) | 8.0% (6.1% - 10.5%) | 7.1% (5.1% - 9.9%) |
| Suriname | 9.0% (7.5% - 10.8%) | 10.1% (8.2% - 12.3%) | 8.0% (6.2% - 10.4%) |
| Trinidad and Tobago | 15.9% (14.1% - 17.9%) | 19.8% (16.6% - 23.4%) | 12.3% (10.5% - 14.3%) |
| Venezuela | 8.6% (6.7% - 11.1%) | 8.6% (6.2% - 11.9%) | 8.7% (5.8% - 12.8%) |

* The prevalence estimate (P) is standardized by age.
