# Supplementary material for: Prevalence and factors associated with e-cigarettes consumption among adolescents: A cross-sectional analysis in Latin America and the Caribbean
Source: PLOS Glob Public Health. 2026 Aug 11;6(8):e0007058. doi: 10.1371/journal.pgph.0007058 (PMC13460583; doi:10.1371/journal.pgph.0007058)
Supplement: Supplementary file 2 [file pgph.0007058.s002.docx]

**S2 Table.** Factors associated with any and daily use of e-cigarettes: crude and adjusted models using information from 2018 and onwards.

|  | **Any use of electronic cigarettes in the last 30 days** | | **Daily consumption of electronic cigarettes in the last 30 days** | |
| --- | --- | --- | --- | --- |
|  | **Bivariable model** | **Multivariable model** | **Bivariable model** | **Multivariable model** |
|  | **PR (95% CI)** | **PR (95% CI)** | **PR (95% CI)** | **PR (95% CI)** |
| **Sex** |  |  |  |  |
| Female vs. male | 0.87 (0.64 – 1.18) | 0.88 (0.58 – 1.34) | **0.38 (0.20 – 0.73)** | **0.35 (0.16 – 0.75)** |
| **Age** |  |  |  |  |
| For each additional year | **1.33 (1.23 – 1.45)** | **1.29 (1.18 – 1.43)** | 1.21 (0.91 – 1.61) | 1.26 (0.92 – 1.73) |
| **Spending capacity** |  |  |  |  |
| Yes vs. no | **1.52 (1.04 – 2.24)** | 1.58 (0.97 – 2.58) | **2.25 (1.17 – 4.32)** | **4.04 (1.95 – 8.39)** |
| **Exposure to smoking at home** |  |  |  |  |
| Yes vs. no | **2.69 (1.85 – 3.93)** | **2.26 (1.44 – 3.53)** | **4.41 (1.81 – 10.76)** | **3.49 (1.53 – 8.00)** |
| **Exposure to smoking at school** |  |  |  |  |
| Yes vs. no | **2.23 (1.86 – 2.69)** | **2.17 (1.76 – 2.66)** | **2.26 (1.05 – 4.84)** | 1.81 (0.95 – 3.46) |

PR = Prevalence ratio; 95% CI = 95% confidence intervals.

The multivariable models were also adjusted for country as a fixed effect. PR of this latter variable is not shown.
