# Supplementary material for: Prevalence and factors associated with e-cigarettes consumption among adolescents: A cross-sectional analysis in Latin America and the Caribbean
Source: PLOS Glob Public Health. 2026 Aug 11;6(8):e0007058. doi: 10.1371/journal.pgph.0007058 (PMC13460583; doi:10.1371/journal.pgph.0007058)
Supplement: Supplementary file 3 [file pgph.0007058.s003.docx]

**S3 Table.** Factors associated with any and daily use of e-cigarettes: crude and adjusted models (considering country coefficients in models)

|  | **Any use of electronic cigarettes in the last 30 days** | | **Daily consumption of electronic cigarettes in the last 30 days** | |
| --- | --- | --- | --- | --- |
|  | **Bivariable model** | **Multivariable model** | **Bivariable model** | **Multivariable model** |
|  | **PR (95% CI)** | **PR (95% CI)** | **PR (95% CI)** | **PR (95% CI)** |
| **Sex** |  |  |  |  |
| Female vs. male | 0.86 (0.64 – 1.16) | 0.88 (0.58 – 1.31) | **0.38 (0.20 – 0.72)** | **0.35 (0.17 – 0.73)** |
| **Age** |  |  |  |  |
| For each additional year | **1.33 (1.23 – 1.44)** | **1.29 (1.17 – 1.41)** | 1.22 (0.92 – 1.60) | 1.25 (0.92 – 1.70) |
| **Spending capacity** |  |  |  |  |
| Yes vs. no | **1.52 (1.04 – 2.21)** | 1.57 (0.97 – 2.54) | **2.22 (1.17 – 4.23)** | **4.01 (1.94 – 8.27)** |
| **Exposure to smoking at home** |  |  |  |  |
| Yes vs. no | **2.68 (1.86 – 3.86)** | **2.25 (1.46 – 3.46)** | **4.40 (1.83 – 10.53)** | **3.47 (1.54 – 7.80)** |
| **Exposure to smoking at school** |  |  |  |  |
| Yes vs. no | **2.23 (1.86 – 2.67)** | **2.16 (1.77 – 2.62)** | **2.28 (1.08 – 4.82)** | 1.83 (0.98 – 3.43) |
| **Country (vs. Venezuela)** |  |  |  |  |
| Antigua & Barbuda | **0.48 (0.34 – 0.68)** | **0.39 (0.27 – 0.58)** | **0.37 (0.15 – 0.88)** | **0.21 (0.08 – 0.57)** |
| Argentina | 0.93 (0.60 – 1.43) | 0.88 (0.58 – 1.34) | 0.58 (0.22 – 1.52) | 0.47 (0.18 – 1.21) |
| Belize | **0.71 (0.52 – 0.97)** | **0.64 (0.45 – 0.93)** | 0.55 (0.24 – 1.24) | **0.42 (0.18 – 0.99)** |
| Bolivia | 0.93 (0.69 – 1.25) | 0.80 (0.58 – 1.10) | 0.47 (0.21 – 1.05) | **0.37 (0.15 – 0.88)** |
| Cuba | 0.68 (0.45 – 1.04) | **0.55 (0.37 – 0.81)** | **0.48 (0.24 – 0.99)** | **0.35 (0.16 – 0.78)** |
| El Salvador | **0.07 (0.04 – 0.14)** | **0.08 (0.04 – 0.15)** | **0.05 (0.01 – 0.24)** | **0.05 (0.01 – 0.21)** |
| Jamaica | **1.38 (1.08 – 1.77)** | 1.07 (0.78 – 1.47) | 0.67 (0.39 – 1.17) | **0.47 (0.23 – 0.94)** |
| Nicaragua | 0.93 (0.69 – 1.26) | 0.81 (0.56 – 1.17) | -- | -- |
| Panama | 0.75 (0.54 – 1.04) | 0.80 (0.55 – 1.16) | **0.04 (0.01 – 0.32)** | **0.04 (0.00 – 0.28)** |
| Paraguay | 1.30 (0.98 – 1.73) | **1.60 (1.17 – 2.18)** | **0.52 (0.28 – 0.97)** | **0.49 (0.28 – 0.86)** |
| Peru | 0.76 (0.56 – 1.02) | **0.70 (0.50 – 0.98)** | **0.10 (0.03 – 0.29)** | **0.08 (0.03 – 0.24)** |
| St Lucia | 1.19 (0.85 – 1.66) | 1.23 (0.85 – 1.77) | 0.73 (0.33 – 1.60) | 0.58 (0.27 – 1.23) |
| St Vincent & Grenadines | 0.88 (0.63 – 1.22) | 0.70 (0.47 – 1.04) | 0.69 (0.30 – 1.57) | 0.42 (0.16 – 1.08) |
| Suriname | 1.04 (0.77 – 1.40) | 0.99 (0.71 – 1.38) | 0.61 (0.26 – 1.41) | 0.49 (0.19 – 1.29) |
| Trinidad & Tobago | **1.84 (1.40 – 2.41)** | **1.53 (1.10 – 2.14)** | 1.84 (0.92 – 3.69) | 1.19 (0.56 – 2.52) |

PR = Prevalence ratio; 95% CI = 95% confidence intervals. Venezuela was chosen as the reference category due to its intermediate prevalence, and large sample to ensure estimates stability.
