# Supplementary material for: Prevalence and factors associated with e-cigarettes consumption among adolescents: A cross-sectional analysis in Latin America and the Caribbean
Source: PLOS Glob Public Health. 2026 Aug 11;6(8):e0007058. doi: 10.1371/journal.pgph.0007058 (PMC13460583; doi:10.1371/journal.pgph.0007058)
Supplement: Supplementary file 4 [file pgph.0007058.s005.docx]

Peer Review — Manuscript PGPH-D-26-00152R1

Title: Prevalence and factors associated with e-cigarette consumption among adolescents: a cross-sectional analysis in Latin America and the Caribbean

General Assessment

This revised manuscript addresses an important and underexplored public health question: the epidemiology of e-cigarette use among adolescents in Latin America and the Caribbean (LAC). The authors have responded to most previous reviewer comments and the manuscript has improved in clarity and completeness. The study benefits from a large, multi-country sample derived from a standardised surveillance instrument (GYTS), and the analytical choices are generally appropriate. However, several methodological concerns remain that require attention before the manuscript can be recommended for publication. My comments below are intended to be constructive and to strengthen the scientific rigour of the work.

Major Comments

1. Temporal heterogeneity of the pooled dataset (Critical)

The data span eight years (2014–2022), a period during which the e-cigarette market, product diversity, regulatory landscape, and social norms around vaping have changed substantially across the region. Yet the pooled analysis treats observations from 2014 (Belize) and 2022 (Suriname) as if they were contemporaneous. This introduces a major source of heterogeneity that is neither quantified nor adequately discussed. At minimum, the authors should: (a) present the survey year for each country prominently in the results, which they do in Table 1, and (b) include survey year as a covariate in the multivariate models, or at least conduct a sensitivity analysis excluding surveys administered before 2018 (the approximate period of rapid market expansion). The absence of any temporal trend analysis or adjustment is a significant oversight that limits the interpretability of the pooled estimates.

2. High proportion of missing data in El Salvador (Critical)

El Salvador presents 34.4% missing values on the variables of interest - by far the highest in the dataset and well above conventional thresholds for concern (typically >10–20%). The authors acknowledge this in Table 1 but offer no sensitivity analysis, no imputation strategy, and no examination of whether the missing data pattern is random or systematic (i.e., whether missingness is associated with e-cigarette use or key covariates). Given that El Salvador also reports the lowest prevalence of e-cigarette use in the entire sample (0.6%), one must ask whether this figure reflects a true epidemiological reality or is driven by selection bias from the high non-response. This country should either be excluded from the main analysis and treated as a sensitivity scenario, or a formal missing data analysis (e.g., missing at random assumption test, multiple imputation, or complete case analysis with formal comparison) should be conducted and reported.

3. Inconsistency in sample size reporting

The manuscript states in the Results section that data from 55,330 adolescents were analysed, yet Table 1 is headed "Data available for statistical analysis (n = 50,729)." This discrepancy of approximately 4,600 participants is unexplained. The authors must clarify which figure represents the analytical sample and reconcile these numbers throughout the manuscript, including in the abstract.

4. Operationalisation of daily e-cigarette use

The authors define daily use as using e-cigarettes on all 30 of the past 30 days. This is a highly restrictive threshold that likely underestimates the true burden of habitual or dependent use. Standard epidemiological practice typically defines daily use as use on 20 or more days within the past 30 days, or at least "most days." Under the authors' definition, an adolescent who vaped 29 out of 30 days would be categorised as a non-daily user. The authors should justify this choice with reference to published literature or standard surveillance definitions, and consider reporting estimates using the more conventional ≥20 days/month threshold as a sensitivity analysis, particularly given the public health implications of their conclusions regarding nicotine dependence.

5. Pooling without accounting for between-country heterogeneity

While the complex survey design (strata, clusters, and weights) is appropriately accounted for within each country, the pooled multi-country analysis does not appear to incorporate country as a level in the model (e.g., through multilevel or mixed-effects Poisson regression, or through the inclusion of country fixed effects). This is important because observations from the same country share unmeasured contextual characteristics (regulatory environment, cultural norms, product availability), and ignoring this clustering may lead to underestimated standard errors and inflated statistical significance. The authors should either include country-level fixed effects in the multivariate model or explicitly justify why this was not done given the design.

6. Justification for Poisson regression

The authors state they opted for Poisson regression to avoid overestimation of associations when outcome prevalence exceeds 10%. However, the overall pooled prevalence is 8.0%, which falls below this threshold. The justification is valid for several individual countries (e.g., Trinidad and Tobago at 15.9%), but the pooled prevalence does not meet the stated criterion. The authors should either revise their rationale (e.g., noting that country-specific prevalences in several settings exceed 10% and that the pooled model must accommodate this heterogeneity) or provide a supplementary logistic regression analysis for transparency.

Minor Comments

7. Exposure window inconsistency across covariates

The passive exposure to tobacco smoke at home is assessed over the last 7 days, the exposure at school over the last 30 days, and e-cigarette use over the last 30 days. This inconsistency in the reference periods for exposure and outcome variables is not acknowledged. The shorter reference window for home exposure may attenuate the estimated association and should at minimum be noted as a limitation.

8. Binary operationalisation of spending capacity

The decision to dichotomise spending capacity (any money vs. no money) is understandable given the multi-country, multi-currency design. However, this loses important gradation: an adolescent with token pocket money is categorised identically to one with substantial discretionary income. The authors should discuss how this dichotomisation may affect the estimated association (likely towards the null), and briefly consider whether the GYTS data offer any ordered response categories that might have allowed for a more nuanced analysis.

9. Sex as a binary variable

The manuscript analyses sex as a binary variable (male/female) consistent with the GYTS instrument. The authors should briefly acknowledge that this does not capture gender diversity and that non-binary or gender-nonconforming adolescents may have distinct patterns of e-cigarette use, as has been documented in higher-income settings.

10. Generalisability and country coverage

The manuscript covers 16 countries but excludes major LAC nations including Brazil, Mexico, Colombia, Chile, and Peru (the latter actually appears in Table 1, so Peru is included - but Brazil, Mexico, and Colombia are absent). The authors mention that some countries do not participate in the GYTS, but they should be more explicit about this limitation in terms of representativeness of the findings for the entire LAC region. A brief listing of excluded countries and the reason for their exclusion (e.g., GYTS non-participation) in the Methods section would strengthen transparency.

11. Age standardisation reference population

The WHO standard population used for age standardisation is designed for broad age ranges across an entire population. Its application to a narrow age band of 12–16 year olds is methodologically unusual and should be justified. The authors might consider whether internal standardisation (using the age distribution of the pooled dataset as reference) would be more appropriate and interpretable in this context.

12. Subheadings in the Discussion

Reviewer 2 recommended removing subheadings from the Discussion, and the authors deferred this decision to the editor. While the journal does not strictly prohibit subheadings, it is worth noting that for a manuscript of this length and complexity, subheadings do facilitate readability and navigation, particularly for readers from policy and practice backgrounds. I concur with the authors' choice to retain them and leave the final decision to the editor.

13. Minor textual issues

The abstract concludes that e-cigarette use "is associated with economic factors," yet in the body of the paper, spending capacity is only significantly associated with daily use, not with any use in the past 30 days. This statement in the abstract is therefore misleading and should be revised to accurately reflect the differential findings by outcome.

Line 151: The text states that missing values ranged from 0.1% to "24.4%" in El Salvador, whereas Table 1 shows 34.4% for El Salvador. This appears to be a typographical error.

The Discussion refers to "the lack of intervention studies in the region" (line 241–242), which is a strong claim that is not supported by a systematic search of the literature. The authors should qualify this statement (e.g., "to our knowledge" or "based on available evidence in the GYTS framework").

Reference 8 (Freeman & Chapman, 2009) relates to the Camel cigarette brand and Web 2.0 marketing and seems tangentially related to the cited points about digital marketing and influencers in the context of e-cigarettes. The authors may wish to consider a more directly relevant reference.

Ethical and Data Availability Considerations

The ethical approval is clearly stated, and the data availability statement appropriately directs readers to the WHO NCD Microdata Repository. These aspects are satisfactory.

Summary Recommendation

This manuscript addresses a significant knowledge gap regarding e-cigarette use in LAC adolescents and makes a meaningful contribution to the global surveillance literature. However, publication requires that the authors satisfactorily address the major concerns raised above, particularly: (1) the temporal heterogeneity of the pooled data and its implications for interpretation; (2) the handling of missing data from El Salvador; (3) the discrepancy in reported sample sizes; (4) the definition and threshold for daily use; and (5) the absence of country-level effects in the multivariate model. Addressing these points will substantially strengthen the scientific credibility of the conclusions drawn.

Decision: Major Revision
