# Supplementary material for: Prevalence and factors associated with e-cigarettes consumption among adolescents: A cross-sectional analysis in Latin America and the Caribbean
Source: PLOS Glob Public Health. 2026 Aug 11;6(8):e0007058. doi: 10.1371/journal.pgph.0007058 (PMC13460583; doi:10.1371/journal.pgph.0007058)
Supplement: Supplementary file 5 [file pgph.0007058.s006.docx]

**PGPH-D-26-00152
Prevalence and factors associated with e-cigarettes consumption among adolescents: a cross-sectional analysis in Latin America and the Caribbean**

**Journal Requirements**

**1. Please amend your online Financial Disclosure statement. If you did not receive any funding for this study, please simply state: "The authors received no specific funding for this work."**

***Response:*** We have made changes as requested.

**2. Please update your online Competing Interests statement. If you have no competing interests to declare, please state: "The authors have declared that no competing interests exist."**

***Response:*** We have made changes as requested.

**3. Please provide separate main figure files in .tif or .eps format only and ensure that all files are under our size limit of 10MB. For more information about how to convert your figure files please see our guidelines:** [**https://journals.plos.org/globalpublichealth/s/figures**](https://journals.plos.org/globalpublichealth/s/figures)

***Response:*** We have made changes as requested.

**4. If the reviewer comments include a recommendation to cite specific previously published works, please review and evaluate these publications to determine whether they are relevant and should be cited. There is no requirement to cite these works unless the editor has indicated otherwise.**

**Response:** We have included a couple of new references that are relevant to the text of the manuscript.

**Reviewers' comments:**

**Reviewer #1: Recommendations**

You need to define the target population, more specifically, what age group comprises the data set. Additionally, highlight why this group is important to study, especially in the context of tobacco control.

**Line 47. It may be more useful to state the age of middle school students, as this may vary across countries. Line 47- 48: Please reframe the sentence to make it more coherent - not sure what you’re trying to highlight here.**

***Response:*** The original paper does not define age of each student group as this can be variable. However, they define groups according to grades and report the mean age for each group. We have added that information and rewritten the sentence as suggested by the reviewer. Now it reads: “*In certain high-income countries, such as the United States, the prevalence of EC use within the last 30 days among middle school students (grades 6-8, mean age: 12.7 years) exceeds 25%. However, this estimate was approximately 10% among high school students (grades 9-12, mean age: 16.1 years)*”.

**Line 49-50: It will be useful to understand which countries were towards the higher and lower end.**

***Response:*** We have added the information requested. Now it reads: “*In Latin America, while the literature remains scarce, a systematic review of 14 studies reported an EC use prevalence among adolescents across six countries ranging from 2.4% in Argentina to 64.2% in Mexico, with a combined mean prevalence of 18.9%*”

**Line 57-59: This is an important point; it will be useful to remove the brackets and explain the factors coherently.**

***Response:*** We have rewritten this section as requested, Now it reads: “*The adolescent group is particularly vulnerable to EC use due to a combination of individual factors, such as the perception of low risk; social factors, including peer pressure and the family environment; and contextual factors, such as advertising, availability, local policies*”.

**Line 62: Is there no data on prevalence in the Caribbean at all? Please make that clear.**

***Response:*** The prevalence of EC has been estimated in Puerto Rico, Guatemala, Mexico and Argentina, but no information from the Caribbean exists. Now it reads: “*In Latin America, evidence on EC use among adolescents is limited and scattered, whereas no information is available from the Caribbean*”.

**Line 62: Please clarify: Do you mean general regulation, which includes tobacco products and E-cigarettes, but no specific policies for the latter? See:**

<https://tobaccocontrol.bmj.com/content/26/4/440>.

***Response:*** Thanks for the reference. Based on that, we have rewritten this section as follows: “*Some countries have implemented different regulations to regulate EC use. However, many of them regulate EC based on their tobacco classification. Of the existing countries regulating EC, about a third do not have regulation specifically for EC but instead apply existing tobacco control regulation to these products*”.

**Line 88: What kind of data on advertising, exposure? Similarly, school-related behaviours such as ...**

**Response:** We have added some examples, between brackets, to explain data information requested. Now it reads: “*The questionnaire includes 50 to 70 questions organized into thematic modules that address: prevalence of tobacco use (conventional and electronic), exposure to secondhand smoke, attitudes toward tobacco use, access, advertising exposure (tobacco products on TV, social media, etc.), knowledge about the risks of tobacco, and school-based behaviors (schoolmate smoking, teacher smoking, etc.)*”.

**Line 123 - 24: Always good to report all the variables in the model, even if they are not significant.**

***Response:*** As we explain in the text, we have listed and added all the variables in the model, but only those significant were relevant for discussion. We have rewritten the sentence to clarify that point: “*Given the exploratory nature of the analysis, the multivariate model was constructed by listing all the variables reported in a single model, and only those that were significant (p-value <0.05) were considered relevant for discussion*”.

**Line 153: Lowest amongst both sexes? It’s not very clear.**

***Response:*** We have modified the sentence to clarify this point: “*In that line, the prevalence for both sexes was higher in Trinidad and Tobago, whilst the lowest prevalence for both sexes was observed in El Salvador*”.

**Line 185: Reported “spending money” is quite vague and a bit difficult to follow throughout. Would it be possible to find a more appropriate terminology, such as spending capacity? Also, ensure that you use the same language to refer to these factors in the discussion as well.**

***Response:*** Thanks for the suggestion. We have modified the text of the manuscript to include spending capacity instead of spending money.

**Line 193-195: Not sure what this sentence means, please clarify/rephrase.**

***Response:*** We have rephrased the sentence for clarity. Now it reads: “*Furthermore, although the proportion of adolescents reporting daily e-cigarette use remains low, the prevalence of daily consumption is not trivial, representing a critical public concern towards nicotine dependence and other sequelae*”.

**Line 198: Not sure you should use the term “similar”. Instead of the US, it would make more sense to compare the prevalence with countries that are similar to Latin America in terms of income levels, population, regulatory structure - if any.**

***Response:*** We have modified the term “similar” as suggested. We have also added information from Latin America countries but not related to the GYTS: “*In our analysis, the countries with the highest prevalence of e-cigarette use in the past 30 days were Trinidad and Tobago (15.9%), Jamaica (12.5%), and Paraguay (11.3%). These values fall within the range reported by a recent regional analysis of Latin American adolescents, which documented prevalences between 2.4% and 64.2%. Mexico (64.2%), Colombia (32.3%), and Puerto Rico (21.9%) showed the highest prevalence, but these countries do not collect data in the GYTS*”.

**Line 205-206: Where is the data from then?**

***Response:*** We have added the information requested: “*In different cross-sectional surveys, Mexico (64.2%), Colombia (32.3%), and Puerto Rico (21.9%) showed the highest prevalence of EC use, but these countries do not collect data in the GYTS*”.

***You may consider strengthening the Discussion section to make it more relevant. For instance, what are the implications for policy, practice (programs), and future research? Questions that could help anchor your discussion include: What existing regulations and policies are in place in the region, and how can your data inform the development or refinement of policy? What does partial regulation comprise?***

***Response:*** We have rewritten the “Relevance of the findings” section to make it more relevant and clearer.

**Are there examples of school-based or family interventions that have worked in the region?**

***Response:*** Unfortunately, this kind of examples are not from the region, although many of them are from the US, a high-income country, not comparable to Latin American region. We have added a sentence regarding this point: “*Moreover, the lack of intervention studies in the region suggests the need to adapt or use strategies used in high-income countries*”.

**Reviewer #2: Recommendations**

**The health effects of e-cigarettes can be provided in introduction.**

Response: We have added some of the deleterious health effects of using EC: “*The use of electronic cigarettes (e-cigarettes or EC) has increased significantly over the last decade, emerging as a major challenge for global public health, with potential health complications, including respiratory irritation, lung edema, sustained tissue injury, arterial stiffness, among others*”.

**The reason for using Poisson regression over logistic regression may be explained.**

***Response:*** We have added the explanation of using Poisson instead of logistic regression. We have also added a reference regarding this point: “We opted for Poisson instead of logistic regression because strength of association may be overestimated when the prevalence of the outcome (EC use) exceeds 10% as reported in many countries (overestimation bias) [a]”.

***Reference:***

a. Barros AJ, Hirakata VN. Alternatives for logistic regression in cross-sectional studies: an empirical comparison of models that directly estimate the prevalence ratio. BMC Med Res Methodol 2003;3:21

**Discussion need not have subheadings such as main finding, comparison with previous studies etc.**

***Response:*** I leave this decision to the editor. The format of the journal allows us to use different levels of subheading, for instance, we believe using them is acceptable.
