# Supplementary material for: Prevalence and factors associated with e-cigarettes consumption among adolescents: A cross-sectional analysis in Latin America and the Caribbean
Source: PLOS Glob Public Health. 2026 Aug 11;6(8):e0007058. doi: 10.1371/journal.pgph.0007058 (PMC13460583; doi:10.1371/journal.pgph.0007058)
Supplement: Supplementary file 6 [file pgph.0007058.s007.docx]

**Peer Review Report - Round 2**

*PLOS Global Public Health*

Manuscript ID: PGPH-D-26-00152R2

*Title: Prevalence and factors associated with e-cigarette consumption among adolescents: a cross-sectional analysis in Latin America and the Caribbean*

# 1. Overall assessment

The authors have engaged constructively with the previous round of review and have responded to nearly all of my Round 1 comments. The manuscript is now appreciably stronger, particularly in three respects: (i) the temporal heterogeneity of the pooled dataset is partially addressed through the inclusion of country as a fixed effect (which is collinear with survey year given that each country contributes a single survey wave) and a complementary sensitivity analysis restricted to surveys conducted from 2018 onward; (ii) the discrepancy in sample sizes has been reconciled (n = 52,940 after exclusion of 2,390 records with missing information on e-cigarette use); and (iii) a more conventional operationalisation of daily use (≥20 days within the past 30) has been added as a sensitivity analysis.

The authors have also strengthened the limitations section by acknowledging the dichotomisation of spending capacity, the binary operationalisation of sex, the inconsistent reference windows across exposure variables, and the impact of missing data in El Salvador. The abstract has been rewritten to differentiate findings by outcome, the conclusion on intervention studies has been appropriately qualified, and reference 8 has been replaced with a more directly relevant citation.

Nevertheless, several substantive issues remain. Most importantly, the prevalence ratios reported in the abstract do not match the values displayed in Table 2 of the revised manuscript, raising serious concern about the internal consistency of the final dataset. In addition, certain methodological clarifications are still warranted in the body of the manuscript, even where they have been adequately handled in the response letter. The points below are intended to be constructive and to bring the manuscript to a publishable standard.

# 2. Assessment against PLOS Global Public Health publication criteria

| **Criterion** | **Assessment** |
| --- | --- |
| 1. Original research | Yes |
| 2. Not previously published | Yes |
| 3. High technical standard; sufficient methodological detail | Largely yes - minor clarifications still needed |
| 4. Conclusions supported by data | Mostly - abstract figures inconsistent with Table 2 |
| 5. Intelligible writing in standard English | Yes - minor editorial polish required |
| 6. Ethics and research integrity | Yes |
| 7. Reporting guidelines and data availability (STROBE) | Yes |

# 3. Adequacy of responses to Round 1 comments

## 3.1 Adequately addressed

- Sample size discrepancy (R1 #3): Table 1 now reports n = 52,940 and the 2,390 records excluded for missing e-cigarette data are explicitly tabulated. The abstract retains the gross figure of 55,330, which is appropriate provided the analytical denominator is clearly stated in the Results.
- Daily-use operationalisation (R1 #4): the ≥20-days threshold has been incorporated as a sensitivity analysis and the small absolute difference (0.7% vs. 0.9%) is reported. This adequately addresses the previous concern.
- Country-level clustering (R1 #5): country has been entered as a fixed effect in the multivariable Poisson models. The authors note collinearity between country and survey year and have therefore omitted year from the main model, retaining the post-2018 restriction as a sensitivity analysis. This is a reasonable analytical choice.
- Poisson vs. logistic regression (R1 #6): the sideTby-side comparison provided in the response letter is reassuring. Estimates obtained by the two approaches are concordant, with the modest divergence expected when prevalence is moderate. Retention of Poisson regression is justified.
- Exposure-window inconsistency (R1 #7), dichotomisation of spending capacity (R1 #8), binary sex (R1 #9), generalisability (R1 #10), and the strong claim about intervention studies (minor #13): all appropriately reflected in the revised text.
- Replacement of reference 8: the substitution of Vassey et al. (Tobacco Control, 2023) for the previous Freeman & Chapman reference is a clear improvement and better supports the claim about social-media-driven exposure.

## 3.2 Partially addressed — further clarification required

***3.2.1 Temporal heterogeneity (R1 #1)***

The argument that country and survey year are collinear is correct, since each country contributes a single survey wave. However, this important methodological fact is presently confined to the response letter; the manuscript itself does not explain to the reader why year is not in the model. I recommend that the authors add a sentence to the Statistical Analysis section along the lines of: "Because each participating country contributed data from a single survey year, country and survey year were highly collinear; we therefore included country as a fixed effect, which absorbs both unmeasured country-level contextual factors and the time of data collection." Without this clarification, the reader may assume that secular trends are uncontrolled.

In addition, the S2 Table sensitivity analysis restricted to surveys conducted from 2018 onward should be summarised briefly in the Results (e.g., "point estimates and 95% confidence intervals were essentially unchanged") rather than only referenced. Currently the manuscript states only that there were "no changes," which is insufficiently informative for the reader who cannot inspect the supplement.

***3.2.2 Missing data in El Salvador (R1 #2)***

The authors compared pooled prevalences with and without El Salvador and report that estimates varied by only 0.1%. This is reassuring at the pooled level but does not address the more substantive concern: namely, whether the country-specific estimate for El Salvador (0.6%) — by far the lowest in the dataset — is itself trustworthy given that more than one-third of eligible records were excluded. I recommend that the authors briefly examine whether non-response in El Salvador is differential by age or sex (which can be tested directly within their existing dataset) and report this in either the main text or the supplement. A single sentence such as "non-response was [or was not] differentially distributed by age and sex" would resolve the residual concern. The current wording ("results varied only 0.1% (data not shown)") is too terse for a finding of this importance.

***3.2.3 Age standardisation (R1 #11)***

The response that two WHO standard age groups (10–14 and 15–19) were used is reasonable but is not stated in the manuscript. The Methods say only "WHO standard population [18]." Please specify the exact age categories used for direct standardisation, as readers will reasonably ask why a population standard designed for life-course comparisons is applied to a 12–16-year-old sample.

***3.2.4 Justification of Poisson regression in the manuscript text***

Although the response letter persuasively justifies the choice of Poisson regression, the manuscript text still motivates it solely based on an overall pooled prevalence of 8%. Because several country-specific prevalences exceed 10% (e.g., Trinidad and Tobago, Jamaica, Paraguay) and because the pooled model must accommodate this heterogeneity, the rationale would be more accurately stated as follows: "Because country-specific prevalences in several settings exceeded 10%, and because logistic odds ratios would be inflated relative to prevalence ratios in those settings, we used Poisson regression with robust variance." A brief sentence to that effect would close the gap between the response letter and the Methods.

# 4. New issues identified in this revision

***4.1 Critical - Numerical inconsistency between the abstract and Table 2***

The prevalence ratios reported in the Abstract do not correspond to those displayed in Table 2 of the same revision. Specifically:

- **Abstract:** older age PR = 1.26 (95% CI: 1.16–1.38) — *Table 2 (multivariable, any use): 1.29 (1.17–1.41)*
- **Abstract:** home exposure PR = 2.28 (95% CI: 1.52–3.41) — *Table 2: 2.25 (1.46–3.46)*
- **Abstract:** school exposure PR = 2.21 (95% CI: 1.81–2.71) — *Table 2: 2.16 (1.77–2.62)*
- **Abstract:** daily use × spending capacity PR = 3.00 (95% CI: 1.47–6.15) — *Table 2 (multivariable, daily use): 4.01 (1.94–8.27)*

These discrepancies are not minor rounding differences. They suggest that the abstract reports figures from a previous iteration of the analysis (most likely the model without country fixed effects), whereas Table 2 reports the current adjusted estimates. The authors must reconcile these figures throughout the manuscript before resubmission. This is a critical issue because PLOS Global Public Health Criterion 4 requires that "conclusions are presented in an appropriate fashion and are supported by the data," and at present the abstract and the main results table do not agree.

I would urge the authors to perform a systematic verification of every numerical value in the abstract, results text, Tables 2 and 3, and the supplementary tables, against the underlying STATA output, to ensure full internal consistency in the next revision.

***4.2 Methodological - Visibility of the country fixed-effect adjustment***

Footnote to Table 2 currently reads: "The multivariable models were also adjusted for country as a fixed effect. PR of this latter variable is not shown." While the omission of 16 country coefficients from the printed table is reasonable, an interested reader has no way of knowing whether, for instance, Trinidad and Tobago's elevated prevalence persists after adjustment for the included covariates. I suggest the authors provide a forest plot of the adjusted country-specific PRs (with one country as reference) either in the main text or as a supplementary figure. This would substantially enhance the policy-relevant interpretation of the cross-country variability that they themselves emphasise.

***4.3 Sensitivity analyses - completeness of reporting***

The Results refer to S2 Table for the sensitivity analysis restricted to surveys conducted from 2018 onward, but the manuscript provides no qualitative description of how the estimates changed. A single sentence summarising the direction and magnitude of any differences would help readers who do not access the supplement. The same applies to the El Salvador sensitivity analysis (currently labelled "data not shown" - please show it, either in the supplement or in a single sentence).

***4.4 Use of "sex" versus "gender"***

The manuscript uses "sex" in the Methods (consistent with the GYTS instrument) but "male sex" and "men/women" interchangeably in the Discussion. In light of the limitation now acknowledged regarding gender diversity, I recommend the authors use "male" and "female" (referring to sex as recorded) consistently in the Results, and reserve "men/women" for the Discussion only where it does not introduce ambiguity.

# 5. Minor and editorial points

- Line 175: "In that line" — consider "Accordingly" or "Consistent with this pattern."
- Line 203: "In that sense, the highest prevalence in both sexes was in Trinidad and Tobago" — "In that sense" is awkward; "As above" or "Consistent with the overall pattern" would read more naturally.
- Lines 271–277 (Relevance of our findings): the wording is somewhat overwritten (e.g., "proscription of characterising flavors," "deleterious effects of environmental tobacco smoke (ETS) within domestic environments," "legislative variance appears to be a primary determinant"). The substance is sound, but the register is denser than the rest of the manuscript. A light editorial pass to harmonise tone would be welcome.
- Conclusion (line 301): the current conclusion is brief and somewhat generic. It would be strengthened by a one-sentence quantitative summary of the headline findings (e.g., "Approximately one in twelve adolescents reported recent e-cigarette use, with prevalence reaching nearly 16% in Trinidad and Tobago…") before the policy implications.
- References: please verify the formatting of reference 25 (Reynolds et al., Mol Psychiatry 2025), which is missing volume, issue, and page information in the current draft.

# 6. Strengths of the revised manuscript

It is worth emphasising that this manuscript addresses a genuine and important evidence gap. Adolescent e-cigarette use in Latin America and the Caribbean is poorly characterised in the international literature, and the use of a harmonised surveillance instrument across 16 countries - together with appropriate handling of the complex survey design (strata, clusters, and survey weights), age standardisation, and explicit sensitivity analyses - provides a scientifically rigorous regional baseline. The differentiation between any use and daily use is methodologically appropriate and policy-relevant, and the authors have engaged seriously and in good faith with the previous round of review. With the issues outlined above resolved, the paper will be a useful contribution to the literature on adolescent tobacco-product surveillance in low- and middle-income settings.

# 7. Recommendation

**Minor revision.** The manuscript has improved substantially in this revision and meets most of the PLOS Global Public Health criteria for publication. However, the numerical inconsistency between the abstract and Table 2 (Section 4.1) must be reconciled before acceptance, and a small number of clarifications (Sections 3.2.1 -3.2.4 and 4.2 - 4.4) should be incorporated into the manuscript itself rather than left in the response letter. None of these issues requires new analyses; all can be addressed editorially or by transferring information from the rebuttal into the body of the paper. I would be pleased to re-review a further revision.
