# Supplementary material for: Prevalence and factors associated with e-cigarettes consumption among adolescents: A cross-sectional analysis in Latin America and the Caribbean
Source: PLOS Glob Public Health. 2026 Aug 11;6(8):e0007058. doi: 10.1371/journal.pgph.0007058 (PMC13460583; doi:10.1371/journal.pgph.0007058)
Supplement: Supplementary file 7 [file pgph.0007058.s008.docx]

**Peer Review — Manuscript PGPH-D-26-00152R1**

**Title:** Prevalence and factors associated with e-cigarette consumption among adolescents: a cross-sectional analysis in Latin America and the Caribbean

**General Assessment**

This revised manuscript addresses an important and underexplored public health question: the epidemiology of e-cigarette use among adolescents in Latin America and the Caribbean (LAC). The authors have responded to most previous reviewer comments and the manuscript has improved in clarity and completeness. The study benefits from a large, multi-country sample derived from a standardised surveillance instrument (GYTS), and the analytical choices are generally appropriate. However, several methodological concerns remain that require attention before the manuscript can be recommended for publication. My comments below are intended to be constructive and to strengthen the scientific rigour of the work.

***Response:*** We would like to thank the reviewer for the useful comments to improve our manuscript.

**Major Comments**

**1. Temporal heterogeneity of the pooled dataset (Critical)**

The data span eight years (2014–2022), a period during which the e-cigarette market, product diversity, regulatory landscape, and social norms around vaping have changed substantially across the region. Yet the pooled analysis treats observations from 2014 (Belize) and 2022 (Suriname) as if they were contemporaneous. This introduces a major source of heterogeneity that is neither quantified nor adequately discussed. At minimum, the authors should: (a) present the survey year for each country prominently in the results, which they do in Table 1, and (b) include survey year as a covariate in the multivariate models, or at least conduct a sensitivity analysis excluding surveys administered before 2018 (the approximate period of rapid market expansion). The absence of any temporal trend analysis or adjustment is a significant oversight that limits the interpretability of the pooled estimates.

**Response:** With the idea to verify any temporal heterogeneity of the pooled dataset, we have run again our multivariable models under two different conditions:

a. By including survey year (as continuous variable) as a confounder in the models, and
b. By analysing information of surveys from 2018 and onwards.

Results were very similar compared to original information. Moreover, as requested by the reviewer later on this document, the inclusion of country as a fixed effect in the model (together with survey year and the other variables), found that survey year was highly collinear with country. Based on that, we decided to adjust our models for country instead of survey year.

**2. High proportion of missing data in El Salvador (Critical)**

El Salvador presents 34.4% missing values on the variables of interest - by far the highest in the dataset and well above conventional thresholds for concern (typically >10–20%). The authors acknowledge this in Table 1 but offer no sensitivity analysis, no imputation strategy, and no examination of whether the missing data pattern is random or systematic (i.e., whether missingness is associated with e-cigarette use or key covariates). Given that El Salvador also reports the lowest prevalence of e-cigarette use in the entire sample (0.6%), one must ask whether this figure reflects a true epidemiological reality or is driven by selection bias from the high non-response. This country should either be excluded from the main analysis and treated as a sensitivity scenario, or a formal missing data analysis (e.g., missing at random assumption test, multiple imputation, or complete case analysis with formal comparison) should be conducted and reported.

**Response:** We have explored data from El Salvador as requested by the reviewer. We decided to excluded data from El Salvador in a sensitivity analysis. According to that, the pooled prevalence of any use of EC in the last 30 days increases from 8.0% (95%CI: 6.9% - 9.3%) to 8.1% (6.9% - 9.4%). In the same line, results for the daily use of EC in the last 30 days did not change but only in the confidence intervals (from 0.7% (95%CI: 0.5% - 1.1%) with El Salvador information to 0.7% (95%CI: 0.5% - 1.2%) without that information). As a result, we decided to keep the results as they are. We have, however, recognized the limitation in the appropriate section: “*Third, data from El Salvador showed 34% of missing values, which may impact pooled estimates. Thus, we compared results including and excluding data from El Salvador and results varied only 0.1% (data not shown)*”.

**3. Inconsistency in sample size reporting**

The manuscript states in the Results section that data from 55,330 adolescents were analysed, yet Table 1 is headed "Data available for statistical analysis (n = 50,729)." This discrepancy of approximately 4,600 participants is unexplained. The authors must clarify which figure represents the analytical sample and reconciles these numbers throughout the manuscript, including in the abstract.

**Response:** We recognized the mistake in that section. We have modified Table 1 to show the number of records used in the analysis (n = 52,940). We explained the reason of the difference as follows: “*Of the 55,330 records, 2,390 were not considered because of missing values in e-cigarette variables (pooled estimate: 4.2%, ranging from 0.2% in Panama to 34.4% in El Salvador)*”.

**4. Operationalisation of daily e-cigarette use**

The authors define daily use as using e-cigarettes on all 30 of the past 30 days. This is a highly restrictive threshold that likely underestimates the true burden of habitual or dependent use. Standard epidemiological practice typically defines daily use as use on 20 or more days within the past 30 days, or at least "most days." Under the authors' definition, an adolescent who vaped 29 out of 30 days would be categorised as a non-daily user. The authors should justify this choice with reference to published literature or standard surveillance definitions and consider reporting estimates using the more conventional ≥20 days/month threshold as a sensitivity analysis, particularly given the public health implications of their conclusions regarding nicotine dependence.

**Response:** We have decided to include a sensitivity analysis to use the definition of daily EC use as suggested by the reviewer. In absolute terms, the change is very small (from 0.7% using the restrictive definition compared to 0.9% using a more relaxed definition. We have added this information in the results section as follows: “*In sensitivity analysis, using the second definition of daily EC use in the last 30 days, the overall prevalence estimate was 0.9% (95%CI: 0.7%–1.3%), with a higher prevalence in men (1.3%) than women (0.5%, p<0.001)*”.

**5. Pooling without accounting for between-country heterogeneity**

While the complex survey design (strata, clusters, and weights) is appropriately accounted for within each country, the pooled multi-country analysis does not appear to incorporate country as a level in the model (e.g., through multilevel or mixed-effects Poisson regression, or through the inclusion of country fixed effects). This is important because observations from the same country share unmeasured contextual characteristics (regulatory environment, cultural norms, product availability), and ignoring this clustering may lead to underestimated standard errors and inflated statistical significance. The authors should either include country-level fixed effects in the multivariate model or explicitly justify why this was not done given the design.

**Response:** We have adjusted our multivariable models by country as a fixed effect. However, we decided not to include the coefficients for each country in our Table 2. As explained above, survey year was highly collinear with country. Therefore, we decided to keep country as a fixed effect and only show these results excluding survey year before 2018 as a supplementary information.

**6. Justification for Poisson regression**

The authors state they opted for Poisson regression to avoid overestimation of associations when outcome prevalence exceeds 10%. However, the overall pooled prevalence is 8.0%, which falls below this threshold. The justification is valid for several individual countries (e.g., Trinidad and Tobago at 15.9%), but the pooled prevalence does not meet the stated criterion. The authors should either revise their rationale (e.g., noting that country-specific prevalences in several settings exceed 10% and that the pooled model must accommodate this heterogeneity) or provide a supplementary logistic regression analysis for transparency.

**Response:** We have run our models using Poisson and logistic regression. Results are shown in tables below. Despite results did not markedly change, as notice, point estimates with logistic regression (OR) tend to be a bit higher than those using Poisson regression (PR). This is especially important when any use of EC in the last 30 days is the outcome (see below for multivariable models).

| **Any use of electronic cigarettes in the last 30 days (multivariable models)** | | |
| --- | --- | --- |
|  | **PR (95% CI)** | **OR (95% CI)** |
| **Sex** |  |  |
| Female vs. male | 0.88 (0.58 – 1.31) | 0.86 (0.54 – 1.36) |
| **Age** |  |  |
| For each additional year | **1.29 (1.17 – 1.41)** | **1.33 (1.20 – 1.48)** |
| **Spending capacity** |  |  |
| Yes vs. no | 1.57 (0.97 – 2.54) | 1.67 (0.97 – 2.86) |
| **Exposure to smoking at home** |  |  |
| Yes vs. no | **2.25 (1.46 – 3.46)** | **2.53 (1.55 – 4.14)** |
| **Exposure to smoking at school** |  |  |
| Yes vs. no | **2.16 (1.77 – 2.62)** | **2.36 (1.89 – 2.93)** |

Models also adjusted for country (fixed effect). Coefficients are not shown.

| **Daily consumption of electronic cigarettes in the last 30 days (multivariable models)** | | |
| --- | --- | --- |
|  | **PR (95% CI)** | **OR (95% CI)** |
| **Sex** |  |  |
| Female vs. male | **0.35 (0.17 – 0.73)** | **0.34 (0.15 – 0.75)** |
| **Age** |  |  |
| For each additional year | 1.25 (0.92 – 1.70) | 1.26 (0.92 – 1.74) |
| **Spending capacity** |  |  |
| Yes vs. no | **4.01 (1.94 – 8.27)** | **4.12 (1.94 – 8.74)** |
| **Exposure to smoking at home** |  |  |
| Yes vs. no | **3.47 (1.54 – 7.80)** | **3.58 (1.51 – 8.48)** |
| **Exposure to smoking at school** |  |  |
| Yes vs. no | 1.83 (0.98 – 3.43) | 1.86 (0.96 – 3.58) |

Models also adjusted for country (fixed effect). Coefficients are not shown.

Based on this information, we decided to keep our results using Poisson regression models as they are probably not overestimated compared to those using logistic regression.

**Minor Comments**

**7. Exposure window inconsistency across covariates**

The passive exposure to tobacco smoke at home is assessed over the last 7 days, the exposure at school over the last 30 days, and e-cigarette use over the last 30 days. This inconsistency in the reference periods for exposure and outcome variables is not acknowledged. The shorter reference window for home exposure may attenuate the estimated association and should at minimum be noted as a limitation.

**Response:** Thanks for noticing this. We have added it as a limitation as follows: “*Fourth, some temporal inconsistency is found in some variables (i.e., tobacco smoke at home is evaluated over the last 7 days, the exposure at school over the last 30 days, and EC use over the last 30 days). This inconsistency may have effect on some estimates. For example, the shorter reference window for smoke home exposure may have attenuated the point estimate of the association*”.

**8. Binary operationalisation of spending capacity**

The decision to dichotomise spending capacity (any money vs. no money) is understandable given the multi-country, multi-currency design. However, this loses important gradation: an adolescent with token pocket money is categorised identically to one with substantial discretionary income. The authors should discuss how this dichotomisation may affect the estimated association (likely towards the null) and briefly consider whether the GYTS data offer any ordered response categories that might have allowed for a more nuanced analysis.

**Response:** Yes, GYTS information offer an ordered response category. However, as we explained, the multi-currency design of that particular question made difficult to deal with that variable. Dichotomization of spending capacity was the only possible strategy but may affect results. Thus, a dose-response analysis is not possible as pooled data. We have added information regarding this point in different sections of the manuscript:

In Methods: “*The response options had different ordered categories (i.e., from “Do not have any spending money” to greater categories [less than 5 PEN, or greater than 50 PEN in the case of Peru])*”.

In the Limitations: “*Sixth, the dichotomization of the spending capacity variable (no vs. yes) may affect our capacity to show a dose-response analysis as we decided for this strategy to pool multi-country, multi-currency data*”.

**9. Sex as a binary variable**

The manuscript analyses sex as a binary variable (male/female) consistent with the GYTS instrument. The authors should briefly acknowledge that this does not capture gender diversity and that non-binary, or gender-nonconforming adolescents may have distinct patterns of e-cigarette use, as has been documented in higher-income settings.

**Response:** We have added this limitation as follows: “*Fifth, gender diversity was not captured by the GYTS as non-binary or gender-nonconforming adolescents may have distinct patterns of EC use as has been documented in other countries* [a].”

**Reference:**

[a] Felner JK, Andrzejewski J, Strong D, Kieu T, Ravindran M, Corliss HL. Vaping Disparities at the Intersection of Gender Identity and Race/Ethnicity in a Population-Based Sample of Adolescents. Nicotine Tob Res 2022;24(3):349-57.

**10. Generalisability and country coverage**

The manuscript covers 16 countries but excludes major LAC nations including Brazil, Mexico, Colombia, Chile, and Peru (the latter actually appears in Table 1, so Peru is included - but Brazil, Mexico, and Colombia are absent). The authors mention that some countries do not participate in the GYTS, but they should be more explicit about this limitation in terms of representativeness of the findings for the entire LAC region. A brief listing of excluded countries and the reason for their exclusion (e.g., GYTS non-participation) in the Methods section would strengthen transparency.

**Response:** We have added requested information in the Methods section: “*However, some major nations (i.e., Brazil, Mexico, Colombia and Chile) do not participate in the GYTS, which may affect representativeness of our results*”.

**11. Age standardisation reference population**

The WHO standard population used for age standardisation is designed for broad age ranges across an entire population. Its application to a narrow age band of 12–16 year olds is methodologically unusual and should be justified. The authors might consider whether internal standardisation (using the age distribution of the pooled dataset as reference) would be more appropriate and interpretable in this context.

**Response:** We agree with the reviewer, but we have used the two standard groups of WHO population for that (young adolescents:10-14 and older adolescents:15-19 years), which we believe appropriately cover our study group.

**12. Subheadings in the Discussion**

Reviewer 2 recommended removing subheadings from the Discussion, and the authors deferred this decision to the editor. While the journal does not strictly prohibit subheadings, it is worth noting that for a manuscript of this length and complexity, subheadings do facilitate readability and navigation, particularly for readers from policy and practice backgrounds. I concur with the authors' choice to retain them and leave the final decision to the editor.

**Response:** Thanks for the support.

**13. Minor textual issues**

The abstract concludes that e-cigarette use "is associated with economic factors," yet in the body of the paper, spending capacity is only significantly associated with daily use, not with any use in the past 30 days. This statement in the abstract is therefore misleading and should be revised to accurately reflect the differential findings by outcome.

**Response:** We have rewritten the conclusion of the abstract according to reviewer’s comment. Now it reads: “*Among adolescents in Latin America and the Caribbean, e-cigarette use was significant and depended on the indicator used. Any e-cigarette use was associated with older age, smoking exposure at home and at school, whereas daily e-cigarette use was greater among men, and associated with spending capacity and smoking exposure at home. These findings underscore the need to strengthen public policies that restrict access to these products and promote tobacco- and vaping-free school and home environments*”.

**Line 151:** The text states that missing values ranged from 0.1% to "24.4%" in El Salvador, whereas Table 1 shows 34.4% for El Salvador. This appears to be a typographical error.

**Response:** We have corrected this typo. The correct value is 34.4%.

The Discussion refers to "the lack of intervention studies in the region" (line 241–242), which is a strong claim that is not supported by a systematic search of the literature. The authors should qualify this statement (e.g., "to our knowledge" or "based on available evidence in the GYTS framework").

**Response:** We have modified the sentence according to reviewer’s suggestion: “*Moreover, to our knowledge, the lack of intervention studies in the region suggests the need to adapt or use strategies used in high-income countries*”.

Reference 8 (Freeman & Chapman, 2009) relates to the Camel cigarette brand and Web 2.0 marketing and seems tangentially related to the cited points about digital marketing and influencers in the context of e-cigarettes. The authors may wish to consider a more directly relevant reference.

**Response:** We have changed that reference as suggested by the reviewer. We have now included another reference:

- Vassey J, Valente T, Barker J, Stanton C, Li D, Laestadius L, et al. E-cigarette brands and social media influencers on Instagram: a social network analysis. Tob Control 2023;32(e2):e184-e91.

**Ethical and Data Availability Considerations**

The ethical approval is clearly stated, and the data availability statement appropriately directs readers to the WHO NCD Microdata Repository. These aspects are satisfactory.

**Response:** Thanks.

**Summary Recommendation**

This manuscript addresses a significant knowledge gap regarding e-cigarette use in LAC adolescents and makes a meaningful contribution to the global surveillance literature. However, publication requires that the authors satisfactorily address the major concerns raised above, particularly: (1) the temporal heterogeneity of the pooled data and its implications for interpretation; (2) the handling of missing data from El Salvador; (3) the discrepancy in reported sample sizes; (4) the definition and threshold for daily use; and (5) the absence of country-level effects in the multivariate model. Addressing these points will substantially strengthen the scientific credibility of the conclusions drawn.

**Response:** We have modified the manuscript to address all the comments and reviewer’s suggestions.
