# Supplementary material for: Prevalence and factors associated with e-cigarettes consumption among adolescents: A cross-sectional analysis in Latin America and the Caribbean
Source: PLOS Glob Public Health. 2026 Aug 11;6(8):e0007058. doi: 10.1371/journal.pgph.0007058 (PMC13460583; doi:10.1371/journal.pgph.0007058)
Supplement: Supplementary file 8 [file pgph.0007058.s009.docx]

**Peer Review Report - Round 3**

*PLOS Global Public Health*

Manuscript ID: PGPH-D-26-00152R3

*Title: Prevalence and factors associated with e-cigarette consumption among adolescents: a cross-sectional analysis in Latin America and the Caribbean*

# 1. Overall assessment

I have reviewed this manuscript twice previously (Rounds 1 and 2). The authors have engaged seriously and constructively with each round, and the third revision represents a clear and sufficient response to all the substantive concerns I raised previously. Most importantly, the critical numerical inconsistency I flagged in Round 2 - between the prevalence ratios reported in the Abstract and those displayed in Table 2 - has been entirely resolved. I systematically verified all six values; the Abstract, Results text, and Table 2 are now fully internally consistent.

The methodological clarifications I requested have been incorporated into the manuscript itself rather than confined to the response letter: the collinearity between country and survey year is now explicitly explained in the Statistical Analysis (lines 143–145), the WHO standard age groups are specified (line 135), the rationale for Poisson regression is reformulated in terms of country-specific prevalences exceeding 10% (lines 139–141), and the sensitivity analyses are now described qualitatively in the Results. The Discussion has been tightened, the Conclusion now opens with a useful quantitative summary, and the supplementary information has been expanded to include S3 (country fixed-effect coefficients). The authors have also performed and reported an additional analysis of differential non-response in the El Salvador subsample, which I had requested.

In my view, the manuscript now satisfies all seven PLOS Global Public Health publication criteria. Only one substantive point and a small number of editorial details remain, none of which would require a further round of formal revision. I recommend acceptance, with the option for the editors to request minor textual changes during copy-editing if they wish.

# 2. Assessment against PLOS Global Public Health publication criteria

| 1. Original research | Met |
| --- | --- |
| 2. Not previously published | Met |
| 3. High technical standard; sufficient methodological detail | Met |
| 4. Conclusions presented in an appropriate fashion and supported by the data | Met (numerical consistency now verified) |
| 5. Intelligible writing in standard English | Met |
| 6. Ethics and research integrity | Met |
| 7. Reporting guidelines and data availability (STROBE) | Met |

# 3. Resolution of Round 2 comments

| Country/year collinearity not explained in manuscript | Explicit sentence added to Statistical Analysis (lines 143-145) | Resolved |
| --- | --- | --- |
| S2 sensitivity analysis only referenced, not described | Qualitative summary added in Results ("point estimates and 95% CI essentially unchanged") | Resolved |
| El Salvador non-response not tested for differential distribution | Tested and reported: not differential by sex, but differential by age group | Largely resolved (see 3.1) |
| WHO standard age groups not specified | "10-14 and 15-19 years" now stated explicitly (line 135) | Resolved |
| Poisson rationale based on pooled prevalence, not country-specific | Rewritten to reference country-specific prevalences exceeding 10% (lines 139-141) | Resolved |
| **CRITICAL: Abstract PRs did not match Table 2** | **All six values now reconciled and verified against Table 2** | **Fully resolved** |
| Country fixed-effect coefficients not visible to reader | Added as S3 Table (Venezuela as reference) | Resolved |
| Inconsistent use of "sex" vs "gender" / "men-women" | Results use "males/females"; Discussion uses "men/women" appropriately | Resolved |
| Stylistic points (lines 175, 203; over-dense Relevance section; brief Conclusion; ref. 25) | All addressed: rephrasing, tone harmonisation, quantitative summary added to Conclusion, reference 25 now complete | Resolved |

## 3.1 The one remaining substantive point - implication of differential non-response by age in El Salvador

In response to my Round 2 request, the authors tested whether non-response in El Salvador was differential by age and sex and now report (lines 294–296) that it was not differential by sex but was differential by age group. I welcome this analysis. However, the manuscript states the finding without discussing its implications, which deserve a brief sentence.

The 30-day prevalence of e-cigarette use rises with age (this is one of the headline findings of the paper itself), and the analysis excluded approximately one-third of eligible El Salvador records on the e-cigarette variables. If older adolescents — who are more likely to vape — were preferentially among the missing, this could plausibly bias the El Salvador prevalence estimate downward, which is consistent with the observation that El Salvador has the lowest prevalence in the entire dataset (0.6%). I would suggest adding a single sentence to the limitations, for example: "Because non-response in El Salvador was differential by age group, and because e-cigarette use rises with age in our data, the country-specific estimate for El Salvador may be biased downward; pooled estimates were unaffected as confirmed by the sensitivity analysis." This would acknowledge the most plausible direction of bias without overstating it.

I want to emphasise that this is a clarification, not a methodological objection. The transparent reporting of the finding by the authors is already a substantial improvement and meets the standard of intellectual honesty expected by the journal.

# 4. Remaining minor editorial points

- Strengths section (lines 285–288): the sentence "Adolescent use of e-cigarettes in Latin America and the Caribbean is poorly characterized in international literature, and the use of a harmonized surveillance instrument across 16 countries provides a scientifically rigorous regional baseline" partly duplicates content already present in the Introduction and Discussion. It could be condensed or removed without loss of substance.
- Relevance of our findings (lines 269–281): the editorial pass requested in Round 2 has improved this section, but a few formulations remain denser than the surrounding text — notably "prohibition of flavored cigarettes" (consider "banning flavoured e-cigarette products," since flavoured combustible cigarettes are a different regulatory issue), "legislative variability may serve as a critical driver," and "positive correlation with elevated prevalence rates among adolescent cohorts." A light copy-edit at proof stage would suffice.
- **S3 Table - choice of reference country:** the authors have chosen Venezuela as the reference category for the country fixed effect. This is a defensible choice (intermediate prevalence, large sample), but it could be useful to note in the table footnote *why* Venezuela was selected, so that readers can interpret the country-specific PRs without ambiguity.
- Line 208: "the lowest was in Nicaragua (0.0%, 95% CI: 0.0%–0.04%)." The upper bound "0.04%" should probably read "0.4%" or, if it is indeed 0.04%, the CI is given to one more decimal place than the point estimate — please verify and harmonise the precision.
- Keywords: the journal capitalises keywords inconsistently across versions ("Smoking; Prevalence" appear capitalised in the cover page metadata but lower-case in the manuscript itself). Editorial Manager will normalise this, but it is worth checking at proof stage.

# 5. Strengths of the final version

Following three rounds of revision, this manuscript now provides a useful and methodologically sound regional baseline on adolescent e-cigarette use across 16 Latin American and Caribbean countries - a setting that, as the authors correctly emphasise, has been chronically under-represented in the international surveillance literature. The strengths that emerge most clearly in this final version are: (i) appropriate handling of the complex GYTS survey design (strata, clusters, and weights) throughout the analysis; (ii) the explicit differentiation between any use and daily use, with internally consistent estimates for both; (iii) the use of a country fixed effect that absorbs unmeasured country-level confounding and time-of-survey effects, with the underlying methodological reasoning now transparently stated; (iv) a layered set of sensitivity analyses (post-2018 surveys, alternative daily-use definition, exclusion of El Salvador, country-specific coefficients) that together support the robustness of the headline findings; and (v) a candid and complete limitations section that, with the small addition suggested in Section 3.1, will fully reflect the residual sources of uncertainty in the El Salvador subsample.

# 6. Recommendation

**Accept** (with optional minor editorial revisions handled at copy-editing stage). The manuscript meets all seven PLOS Global Public Health publication criteria. The substantive concerns raised in my previous two rounds of review have been adequately addressed, and the single remaining point (Section 3.1) can be resolved by the addition of one clarifying sentence to the limitations. I do not consider that a further round of formal peer review is necessary, but I would be pleased to verify the final version if the editors wish.

**Declaration of competing interests:**

The reviewer declares no competing interests. The reviewer has no prior collaboration with any of the authors and no financial or professional relationship that could be perceived as biasing this assessment.
