# Supplementary material for: Prevalence and factors associated with e-cigarettes consumption among adolescents: A cross-sectional analysis in Latin America and the Caribbean
Source: PLOS Glob Public Health. 2026 Aug 11;6(8):e0007058. doi: 10.1371/journal.pgph.0007058 (PMC13460583; doi:10.1371/journal.pgph.0007058)
Supplement: Supplementary file 10 [file pgph.0007058.s011.docx]

**Peer Review — Manuscript PGPH-D-26-00152R3**

**Title:** Prevalence and factors associated with e-cigarette consumption among adolescents: a cross-sectional analysis in Latin America and the Caribbean

**Peer Review Report - Round 3**

We would like to thank the reviewer for his comments and detailed review of previous versions with suggestions which have benefited our manuscript. In this document, we will focus only on those comments that require a response or change in our manuscript.

**The one remaining substantive point - implication of differential non-response by age in El Salvador**

In response to my Round 2 request, the authors tested whether non-response in El Salvador was differential by age and sex and now report (lines 294–296) that it was not differential by sex but was differential by age group. I welcome this analysis. However, the manuscript states the finding without discussing its implications, which deserve a brief sentence.

The 30-day prevalence of e-cigarette use rises with age (this is one of the headline findings of the paper itself), and the analysis excluded approximately one-third of eligible El Salvador records on the e-cigarette variables. If older adolescents — who are more likely to vape — were preferentially among the missing, this could plausibly bias the El Salvador prevalence estimate downward, which is consistent with the observation that El Salvador has the lowest prevalence in the entire dataset (0.6%). I would suggest adding a single sentence to the limitations, for example: "Because non-response in El Salvador was differential by age group, and because e-cigarette use rises with age in our data, the country-specific estimate for El Salvador may be biased downward; pooled estimates were unaffected as confirmed by the sensitivity analysis." This would acknowledge the most plausible direction of bias without overstating it.

I want to emphasise that this is a clarification, not a methodological objection. The transparent reporting of the finding by the authors is already a substantial improvement and meets the standard of intellectual honesty expected by the journal.

***Response:*** Thanks for the suggestion. We have added the sentence suggested in the limitations section.

**Remaining minor editorial points**

•	Strengths section (lines 285–288): the sentence "Adolescent use of e-cigarettes in Latin America and the Caribbean is poorly characterized in international literature, and the use of a harmonized surveillance instrument across 16 countries provides a scientifically rigorous regional baseline" partly duplicates content already present in the Introduction and Discussion. It could be condensed or removed without loss of substance.

***Response:*** We have deleted that sentence as suggested.

•	Relevance of our findings (lines 269–281): the editorial pass requested in Round 2 has improved this section, but a few formulations remain denser than the surrounding text — notably "prohibition of flavored cigarettes" (consider "banning flavoured e-cigarette products," since flavoured combustible cigarettes are a different regulatory issue), "legislative variability may serve as a critical driver," and "positive correlation with elevated prevalence rates among adolescent cohorts." A light copy-edit at proof stage would suffice.

***Response:*** We have edited the section as requested.

•	S3 Table - choice of reference country: the authors have chosen Venezuela as the reference category for the country fixed effect. This is a defensible choice (intermediate prevalence, large sample), but it could be useful to note in the table footnote why Venezuela was selected, so that readers can interpret the country-specific PRs without ambiguity.

***Response:*** We have added that information at the footnote as requested:

.

•	Line 208: "the lowest was in Nicaragua (0.0%, 95% CI: 0.0%–0.04%)." The upper bound "0.04%" should probably read "0.4%" or, if it is indeed 0.04%, the CI is given to one more decimal place than the point estimate — please verify and harmonise the precision.

***Response:*** We have verified the result and it is 0.04%, so we have rounded the result to 0.1% to avoid to have 0%.

•	Keywords: the journal capitalises keywords inconsistently across versions ("Smoking; Prevalence" appear capitalised in the cover page metadata but lower-case in the manuscript itself). Editorial Manager will normalise this, but it is worth checking at proof stage.

***Response:*** Our keywords are in lower-case letter.
